# Danshen Improves Survival of Patients With Breast Cancer and Dihydroisotanshinone I Induces Ferroptosis and Apoptosis of Breast Cancer Cells

**DOI:** 10.3389/fphar.2019.01226

**Published:** 2019-10-31

**Authors:** Yu-Shih Lin, Yi-Chia Shen, Ching-Yuan Wu, Ying-Ying Tsai, Yao-Hsu Yang, Yin-Yin Lin, Feng-Che Kuan, Cheng-Nan Lu, Geng-He Chang, Ming-Shao Tsai, Cheng-Ming Hsu, Reming-Albert Yeh, Pei-Rung Yang, I-Yun Lee, Li-Hsin Shu, Yu-Ching Cheng, Hung-Te Liu, Yu-Huei Wu, Yu-Heng Wu, De-Ching Chang

**Affiliations:** ^1^Department of Pharmacy, Chiayi Chang Gung Memorial Hospital, Chiayi, Taiwan; ^2^Institute of Molecular Biology, National Chung Cheng University, Chiayi, Taiwan; ^3^Department of Chinese Medicine, Chiayi Chang Gung Memorial Hospital, Chiayi, Taiwan; ^4^School of Chinese Medicine, College of Medicine, Chang Gung University, Tao-Yuan, Taiwan; ^5^Department of Hematology and Oncology, Chiayi Chang Gung Memorial Hospital, Chiayi, Taiwan; ^6^Division of Acupuncture and Chinese Traumatology, Department of TCM, Kaohsiung Chang Gung Memorial Hospital and Chang Gung University College of Medicine, Kaohsiung, Taiwan; ^7^Department of Otolaryngology, Chang Gung Memorial Hospital, Chiayi, Taiwan; ^8^Center of Excellence for Chang Gung Research Datalink, Chang Gung Memorial Hospital, Chiayi, Taiwan

**Keywords:** dihydroisotanshinone I, breast carcinoma, GPX4, National Health Insurance Research Database, danshen, ferroptosis

## Abstract

Danshen (salvia miltiorrhiza Bunge) is widely used in traditional Chinese medicine. However, it is definite clinical effort and mechanism on breast cancer is unclear. In our study, we used the real-world database to investigate *in vivo* protective effort of danshen in the breast cancer patients through using population-based data from the Taiwan National Health Insurance Research Database (NHIRD). *In vitro*, human breast cancer cells (MCF-7 cells and MDA-MB-231 cells) were used to investigate the effect and the underlying mechanism through XTT assay, flow cytometry, glutathione peroxidase (GPX) activity assay, GSH (reduced glutathione)/GSSG (oxidized glutathione), malondialdehyde (MDA), and western blot analysis. The *in vivo* effect was investigated through a xenograft nude mouse model. We found that dihydroisotanshinone I (DT), a pure compound present in danshen, can inhibit the growth of breast carcinoma cells, including MCF-7 cells and MDA-MB-231 cells. Moreover, DT induced apoptosis and ferroptosis in these breast cancer cells. DT also repressed the protein expression of GPX4 (Glutathione peroxidase 4). For *in vivo* study, DT treatment also significantly inhibited the final tumor volume without adverse effects in a xenograft nude mouse model. In conclusion, danshen has protective efforts in breast cancer patients, which could be attributed to DT through inducing apoptosis and ferroptosis of breast cancer cells.

## Introduction

Breast cancer is a common cancer and a common cause of cancer-related death in women in many countries (De Mello Ramirez Medina et al., 2019). The development of new therapeutics for breast cancer has significantly reduced mortality rates, although advanced breast cancer remains an incurable disease. Advanced breast cancer comprises both locally advanced and distant metastatic breast cancer. The median overall survival of patients with metastatic breast cancer is approximately 2–3 years and the 5-year survival rate of patients with metastatic breast cancer is only 25% (Cardoso et al., 2017). Although treatable, remission of breast cancer is often followed by resistance and disease relapse. Therefore, the development of drugs for the treatment of breast cancer remains a research priority.

The National Health Insurance (NHI) program in Taiwan has been reimbursing claims for traditional Chinese medicine, including single herbs or herbal formulae, since 1995. In the previous study, approximately 35.6% of women with breast cancer were treated with traditional Chinese medicine covered by insurance. Moreover, the most frequently used therapies were herbal therapies (80.5%) and acupuncture/traumatology manipulative therapies (22.3%) (Lin and Chiu, 2011). For patients with breast cancer, Lee et al. reported that the use of traditional Chinese medicine was associated with a significantly decreased risk of all-cause mortality through multivariate analysis in NHIRD (Lee et al., 2014). However, the clinical effects and the molecular mechanism of action of herbal therapies for breast cancer are still unclear. Danshen (*salvia miltiorrhiza*) is a commonly used Chinese medicinal herb for the clinical treatment of many types. In our previous studies, we used data from the NHIRD to show that danshen can improve the survival rate of patients with prostate cancer, lung cancer, and colon cancer (Lin et al., 2017; Wu et al., 2017a; Wu et al., 2017b). However, the clinical effects of the traditional Chinese medicine danshen patients on with breast cancer remain unclear.

Ferroptosis is a form of nonapoptotic cell death driven by the loss of activity of the lipid repair enzyme glutathione peroxidase 4 (GPX4) and the subsequent accumulation of lipid-based reactive oxygen species, particularly lipid hydroperoxides (Yang et al., 2014; Yang and Stockwell, 2016). In previous studies, several forms of tanshinones, including tanshinone IIA, acetyltanshinone IIA, and tanshinone I, were found to block the growth of breast cancer cell lines through apoptosis (Nizamutdinova et al., 2008b; Su and Lin, 2008; Tian et al., 2010; Su et al., 2012; Yan et al., 2012; Guerram et al., 2015; Shen et al., 2016). However, the role of ferroptosis in the anti-breast cancer effect of tanshinones remains unclear.

In this study, the NHIRD was used to explore the clinical protective effect of danshen on patients with breast cancer. In addition, we observed that dihydroisotanshinone I (DT) (**Figure 3A**), which is extracted from the dried root of S. miltiorrhiza Bunge, has a significant inhibitory effect on the proliferation of two types breast cancer cell lines, MCF-7 and MDA-MB-231 cells. In the *in vitro* experiments, we discovered that DT induced both apoptosis and ferroptosis in both MCF-7 and MDA-MB-231 cells. Mechanistically, DT could inhibit the protein expression of GPX4. Ferroptosis was subsequently induced through lipid peroxidation. Moreover, DT treatment (30 mg/kg, intraperitoneal injection) significantly inhibited the final tumor volume without inducing adverse effects in nude mice xenografted with tumors. Our results suggest that DT is a novel candidate for future breast cancer treatment.

## Material and Methods

### Data Source

The NHI program of Taiwan, executed in 1995, reimburses both western medicines and traditional Chinese medicine. Nearly the entire 23.7 million populations of Taiwan residents were included into the program by the end of 2010. We used databases for admissions and outpatient visits, both of which included information on patient characteristics such as sex, date of birth, date of admission, date of discharge, dates of visits, and up to five discharge diagnoses or three outpatient visit diagnoses (according to International Classification of Diseases, Ninth Revision (ICD-9) codes) in this study cohort. The data files contained information on patient prescriptions, including the names of prescribed drugs (including western medicines and traditional Chinese medicine), dosage, duration, total expenditure, and other treatment (including operation and radiotherapy). This study adhered to strict confidentiality guidelines according to regulations for personal electronic data protection and was approved by the Ethics Review Board of Chang Gung Memorial Hospital, Chia-Yi Branch, Taiwan (201601433B1).

### Study Subjects

This study cohort was obtained from the Taiwanese National Health Insurance research database, which included all patients who received diagnosis of malignant neoplasm of breast (ICD-9-CM codes: 174) in catastrophic illness database between January 1, 2000, and December 31, 2010. After providing pathological reports or other supporting documents (including pathologic report, laboratory, and image studies), patients who apply for the cancer catastrophic illness certificate are applied to patients. The date of the initial breast cancer diagnosis was defined as the index date of breast cancer. Patients with other cancer diagnosed before breast cancer or missing data were excluded. To confirm the advance (stage III-IV) breast cancer patients in our cohort, we excluded those patients who do not accept taxane, commonly used in stage III-IV breast cancer in Taiwan. In this study cohort, a total of 21,338 patients were included for further study. These patients accrued follow-up time beginning on January 1, 2000, and ended on the date of death, or withdrawal from the registry or on December 31, 2010.

### Danshen Exposure and Potential Confounders and Statistical Analysis for NHIRD

In NHIRD, finished herbal products (FHP), which single herb and herbal formulae are concentrated into granulated compounds, were fully reimbursed under the current NHI system of Taiwan. The list of reimbursed FHP and the drug information, including the proportions of each constituent, date and period of approval as drug, and code and name of manufacturer, was downloaded from the website of the Bureau of NHI. We investigated the original amounts of danshen, in grams, for each mixture of FHPs. In this study, patients were categorized into two groups: had used danshen more than 84 grams after breast cancer diagnosed and those with less than 84 grams danshen used in records. For duration, patients were categorized into two groups: had used danshen more than 28 days after breast cancer diagnosed and those with less than 28 days with treatment of danshen in records. The distribution of demographic factors between the danshen users and nonusers in the study cohort was compared. We used the Kaplan-Meier method to estimate survival probabilities and the log-rank test was performed to examine differences in the risk of death in the cohort. Cox proportional hazards models were used to compute the hazard ratios (HRs) accompanying 95% CIs after adjustment for age, gender, income, urbanization, CCI, and other treatment (operation or radiotherapy). Two-tailed p = 0.05 was considered significant. All of these analyses were conducted using SAS statistical software (Version 9.4; SAS Institute, Cary, NC, USA).

### Cell Culture and Treatment

The human breast cancer cell lines (MCF-7 cells, MDA-MB-231 cells) were obtained from the Bioresource Collection and Research Center. The MCF-7 cells were cultured in Minimum Essential Medium Eagle (Invitrogen Corp., Carlsbad, CA), supplemented with 10% fetal bovine serum at 37°C and 5% CO_2_. The MDA-MB-231 cells were cultured in Leibovitz’s L-15 medium (Invitrogen Corp., Carlsbad, CA), supplemented with 10% fetal bovine serum at 37°C and without CO2 in air. DT was obtained from ChemFaces Natural Products Co., Ltd., China (Catalog number: CFN-90162, the purity of DT is 98% and its solubility in DMSO is >5mg/mL, PubChem CID:89406). Salvianolic acid B (SA) was obtained from what was obtained from Santa Cruz (Catalog number: sc-212911, PubChem CID: 6451084). Oxaliplatin was obtained from Sigma-Aldrich (Catalog number: SI-O9512, PubChem CID: 5310940). Gemcitabine hydrochloride was obtained from Sigma-Aldrich (Catalog number: G6423, PubChem CID:60749). 5-Fluorouracil was obtained from Sigma-Aldrich (Catalog number: F6627, PubChem CID:3385). Before treatment, human breast cancer cells were cultured to 60–70% confluence. Medium was then replaced with fresh medium containing indicated compounds in DMSO (dimethyl sulfoxide) at the indicated concentrations. Treated with DMSO alone was used as untreated vehicle controls.

### XTT Assay

The indicated breast cancer cell lines were plated at a density of 1 × 10^3^ per well, in 96 well plates, in medium containing 10% FBS. Once attached, the medium was replaced with medium containing 10% FBS. The cells were then treated with indicated drugs for indicated hours; and absorbance was measured using the XTT assay kit (Roche, Cat. No. 11465015001) according to the manufacturer’s instructions. The XTT formazan complex was quantitatively measured at 492 nm using an ELISA reader (Bio-Rad Laboratories, Inc.).

### Flow Cytometry

The indicated breast cancer cell lines (1 × 10^6^ cells) were seeded in a 100-mm plate and cultured overnight before treatment. After treated with indicated compounds for indicated hours, the medium was removed and these treated cells were collected. Then, the supernatant was removed by centrifugation, and then resuspended was detected by Annexin V-FITC Apoptosis Detection Kit (Strong Biotech Corporation, Cat No.: AVK250) and Mitoscreen JC-1 kit (BD Biosciences: 551302) according to the manufacturer’s instructions through the flow cytometer BD FACSCanto (Becton Dickinson). Apoptosis of different developmental stages was studied by gating the respective population in the Dot Plots.

### Glutathione Peroxidase (Gpx) Activity Assay

The activity of glutathione peroxidase (GPX) of cell extracts was investigated as described previously (Onaolapo et al., 2017). The cellular extracts of the indicated human breast cancer cell line treated with DMSO or indicated concentrations of drugs for 24 h were prepared according to the manufacturer’s instructions. GPX activity was assessed by measuring the change in absorbance at 340 nm that follow NADPH consumption in the presence of H2O2 that was detected by Glutathione Peroxidase Activity Colorimetric Assay Kit (Bioversion, catalog number: K762-100) according to the manufacturer’s instructions.

### Determination of GSH (Reduced Glutathione)/GSSG (Oxidized Glutathione) and Malondialdehyde (MDA) Levels

The level of GSH (reduced glutathione) and malondialdehyde (MDA) of cell extracts was investigated as described previously (Baysal et al., 2017). The cellular extracts of the indicated human breast cancer cell line treated with DMSO or indicated concentrations of drugs for 24h were prepared according to the manufacturer’s instructions. MDA level was detected by Lipid Peroxidation (MDA) assay kit (Bioversion, catalog number: K739-100) according to the manufacturer’s instructions. GSH (reduced glutathione)/GSSG (oxidized glutathione) level was detected by Glutathione assay kit (Bioversion, catalog number: K264-100) according to the manufacturer’s instructions.

### Western Blot Analysis

For western blotting, cellular extracts after indicated treatments were prepared according to the manufacturer’s instructions. Equal amounts of protein were fractionated on a 10% SDS-PAGE and transferred to polyvinylidene difluoride membranes. The membranes were then blocked with 5% nonfat dried milk for 30 minutes. Then, the membrane was incubated in primary indicated antibody for 6–12 h at room temperature. The list of these primary antibodies was: anti-PARP antibody (Cell Signaling, ratio: 1:1000), anti-GPX4 antibody (proteintech, ratio: 1:1000), and anti-β-actin antibody (Santa Cruz, IB: 1:10000). The primary antibodies and the secondary antibodies were diluted with 1% nonfat dried milk in 0.1% TBST (Tris-Buffered Saline Tween-20). The membranes were washed by 0.1% TBST and incubated in horseradish peroxidase-conjugated secondary antimouse or antirabbit antibodies (Santa Cruz, ratio: 1:5000) for 1 h at room temperature. The protein signal was detected by chemiluminescence, using the Super Signal substrate (Pierce, Number: 34087).

### Mouse Xenograft Model

All procedures involving mouse xenograft model were approved by Animal Care and Use Committee (Approval number 2015060201) of Chang Gung Memory Hospital. Surgery was performed using sodium pentobarbital anesthesia. 10 male BALB/c-nu female nude mice (18–20 g) aged 5–7 weeks were obtained from BioLASCO Taiwan Co., Ltd. and were used to build the xenograft model. In this model, MCF-7 cells were injected (1 × 10^6^/Mouse) subcutaneously in the both flanks of nude mice. Mice with tumor sizes of about 10 mm^3^ were selected after about one week. Mice were randomized into two groups and five mice per each group. One group was treated intraperitoneally with vehicle (2.5% DMSO) and the other one group was treated with 30 mg/kg DT every 2 days. Tumor volume and mouse weight were measured every 2–3 days for 2 weeks. Tumor sizes and tumor volume were calculated using the formula length x width x height x 0.52. Tumor size, body weight, and mortality of the mice were monitored daily. Following 2 weeks, the mice were sacrificed.

### Statistical Analyses

All values were the means ± standard error of mean (SEM) of the replicate samples (n = 3 to 6, depending on the experiment). These experiments were repeated by a minimum of three times. Differences between two groups were assessed using the unpaired two-tailed student’s *t*-test or by ANOVA if more than two groups were analyzed. For testing the significance of pairwise group comparisons, the Tukey test was used as a post-hoc test in ANOVA. *P*-values < 0.05 were considered statistically significant in all comparisons. For all calculations, SPSS version 13.0 for windows (LEAD technologies, Inc.) was used.

## Results

### Protective Effect of Danshen on Patients With Breast Cancer in Taiwan

**Figure 1:**
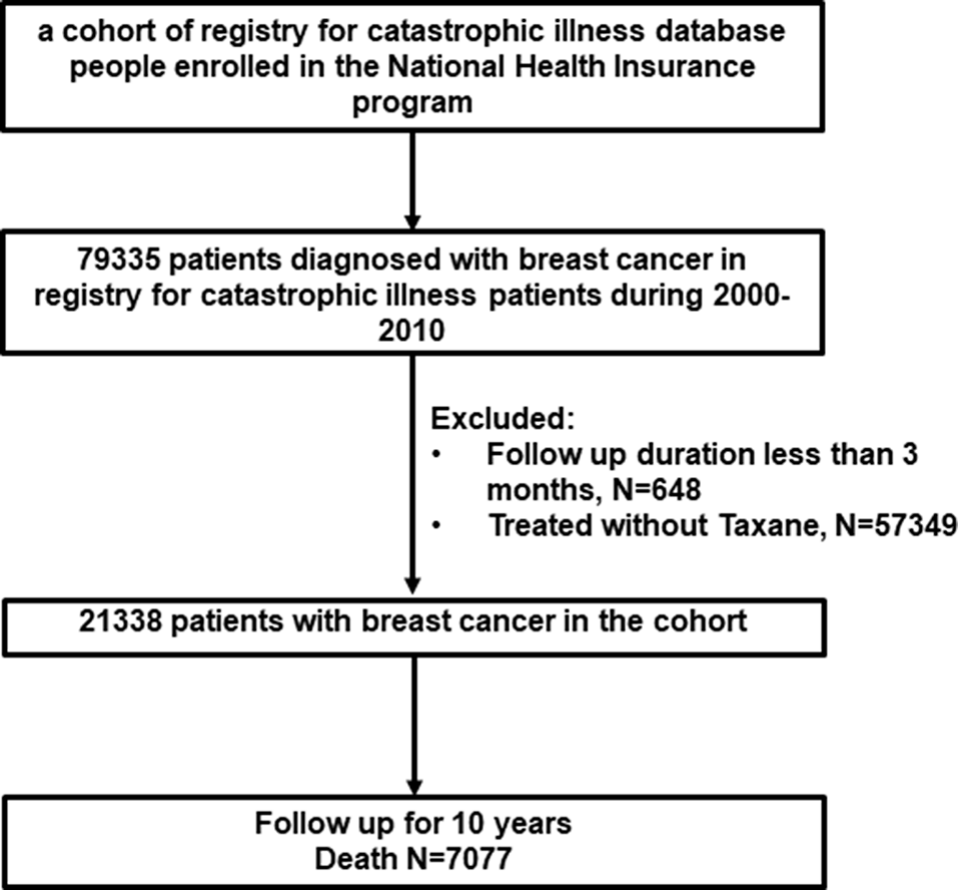
Patient of breast cancer disposition.

**Figure 2:**
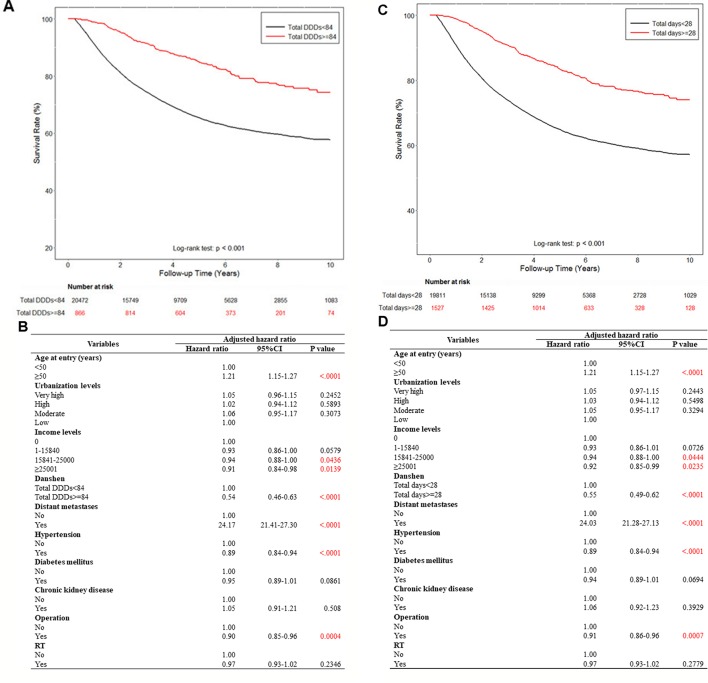
The effect of *danshen* on the survival rate of breast cancer patients in Taiwan. A total of 79,335 breast cancer patients were included in the study cohort. These patients accrued follow-up time for 10 years. Crude overall Kaplan-Meier survival curves for the breast cancer patients was investigated. **(A)** The patients were categorized into 2 groups: never used *danshen*, had used *danshen* more than 84 grams after breast cancer diagnosed, and those with less than 84 grams *danshen* used in records. (log-rank: *p* < 0.001). **(C)** The patients were categorized into two groups: had used danshen more than 28 days after breast cancer diagnosed and those with less than 28 days with treatment of danshen in records (log-rank: *p* < 0.001). **(B**, **D)** Demographic characteristics of breast cancer patients by medications.

This study analyzed a cohort of 79,335 patients with breast cancer between 2000 and 2010. The patient characteristics are summarized in **Figures 2C, D**. As governed by the standard of benefit package of the Taiwan NHI program, only those patients with metastatic breast cancer who did not response to previous combined therapy (stage III–IV) were treated with taxane. Owing to the structure of the NHIRD, we were able to confirm that these patients who were treated with taxane in NHIRD had advanced breast cancer. In this study, we excluded patients who did receive taxane to ensure that the patients in our cohort had stage III–IV disease. After the exclusion of patients who did not meet the inclusion criteria for the study, 21,338 patients remained (**Figure 1**). Highly distant metastases (the adjusted HR of distant metastasis was 24.17 [95% CI, 21.41–27.30] (*p* < 0.001)) in these patients suggested they were patients with breast cancer. In addition, patients with comorbidities, such as diabetes mellitus or chronic kidney disease, were not associated with breast cancer, except hypertension (the adjusted HR of distant metastasis was 0.89 [95% CI, 0.84–0.94] (*p* *<* 0.001)).

We used a cut-off point of 28 days because the limitation of the duration of a single Chinese medicine prescription is 28 days in the Taiwan NHI program; in addition, a previous study also used 28 days as the cut-off point (Yang et al., 2015). In this study, we used a rigorous standard to identify the exposed group by using a 28-day cut-off point. Next, because the average clinical dose of danshen is 3 g per day for clinical Chinese medicine, we used 84 g (3 g for 28 days) as the dose cut-off point. Subsequently, patients were categorized into two groups according to drug dosage after their diagnosis, depending on their medical records: those who had used >84 g of danshen and those who had used ≤84 g of danshen. The patients were also categorized into two groups according to the duration of danshen use after their breast cancer diagnosis: those who had used danshen for >28 days and those who had used danshen for ≤28 days. As danshen is a commonly used Chinese medication and the number of danshen nonusers was very small, danshen nonusers were included into the groups of ≤84 g and ≤28 days. After 10 years, survival rate analysis demonstrated a strong association between the use of danshen and survival (**Figure 2**). By using a multivariate Cox model controlling for age, gender, income, urbanization, Charlson comorbidity index, and other treatment (operation and radiotherapy (RT)), the use of danshen ≥84 g was highly associated with decreased mortality (the adjusted HR of danshen ≥84 g users was 0.54 [95% CI, 0.46–0.63] (*p* <0.001) (**Figures 2A, B**). Moreover, the use of danshen for >28 days remained highly associated with decreased mortality (the adjusted HR of danshen users for >28 days was 0.55 [95% CI, 0.49–0.62] (*p* <0.001) (**Figures 2C, D**). Thus, these data demonstrate the protective effects of a higher dose or longer use of danshen for patients with breast cancer in Taiwan.

### Effect of DT on Growth of Breast Cancer Cells

**Figure 3:**
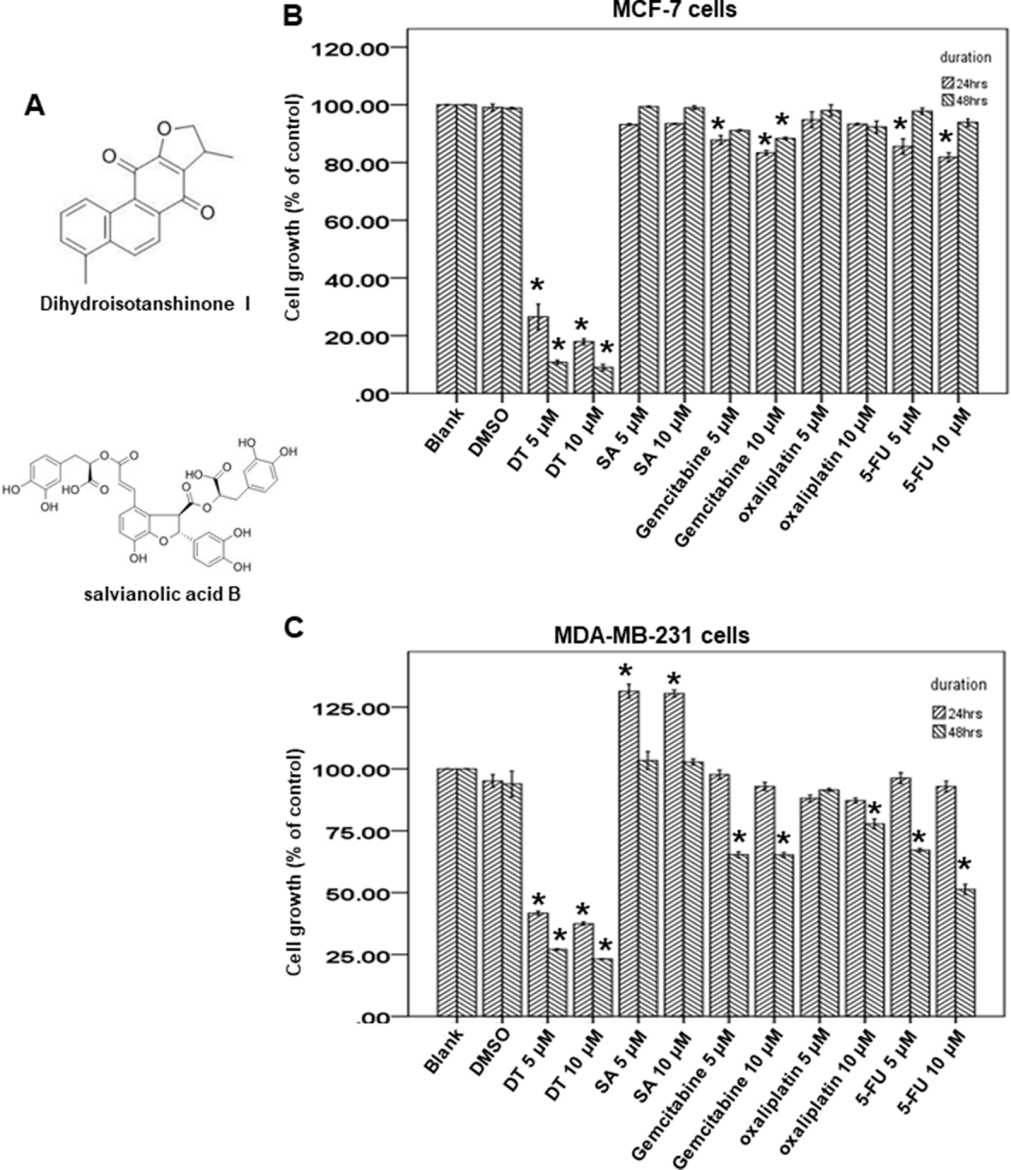
Dihydroisotanshinone I (DT) block the proliferation of breast cancer cell lines. **(A)** The structure of DT and Salvianolic acid B (SA). **(B**, **C)** MCF-7 cells or MDA-MB-231 cells were measured by XTT assay after indicated hours of culturing in the presence of indicated compounds. All the results are representative of at least three independent experiments. (Error bars = mean ± S.E.M. Asterisks (*) mark samples significantly different from DMSO group with *p* < 0.05).

Danshen is a commonly used Chinese medicine in Taiwan and we discovered the clinical protective effect of danshen against patients in Taiwan with breast cancer by using the NHIRD. Next, we aimed to determine which pure compounds in danshen could inhibit the growth of breast cancer cells. The structures of compounds extracted from danshen were divided into two groups: phenolic acids (such as SA) and tanshinones [such as tanshinone IIA, tanshinone 1, and DT (**Figure 3A**)].

Of the tanshinone family, previous studies have demonstrated that tanshinone IIA could induce apoptosis in both ER-positive and ER-negative breast cancers (Wang et al., 2005; Su and Lin, 2008; Su et al., 2012; Yan et al., 2012; Lin et al., 2013; Fu et al., 2014; Li et al., 2015; Li and Lai, 2017; Lin et al., 2018; Lv et al., 2018; Wu et al., 2018). Tanshinone I also exerts a similar anti-breast cancer effect through apoptosis (Nizamutdinova et al., 2008a; Nizamutdinova et al., 2008b; Gong et al., 2012; Wang et al., 2015). As DT belongs to the tanshinone family, DT has a similar structure to tanshinone IIA and tanshinone I. However, the effect and mechanism of DT on breast cancer is unclear; therefore, we investigated the effect of DT on breast cancer.

To investigate the growth inhibitory ability of DT, we used two human breast cell lines, MCF-7 (ER-positive) and MDA-MB-231 cells (ER-negative), as model cells to investigate the effect of SA and DT by using an XTT assay. After treatment with the indicated compounds for 24 to 48 h, 5–10 μM DT significantly inhibited the proliferation of both MCF-7 cells and MDA-MB-231 cells in a dose-dependent and time-dependent manner (**Figures 3B, C**). In addition, DT had a better inhibitory effect than 5–10 μM SA. Notably, 10 μM DT exhibited a stronger inhibitory effect than 10 μM oxaliplatin, gemcitabine, and 5-fluorouracil, commonly used anti-breast cancer clinical agents. Moreover, DT exerted a pronounced inhibitory effect on the proliferation of MCF-7 cells, even at the lowest concentration (5 μM). These data suggest that DT has a role in the growth inhibition of human breast cancer cells.

### Effects of DT on Apoptosis of Breast Cancer Cells *In Vitro*

**Figure 4:**
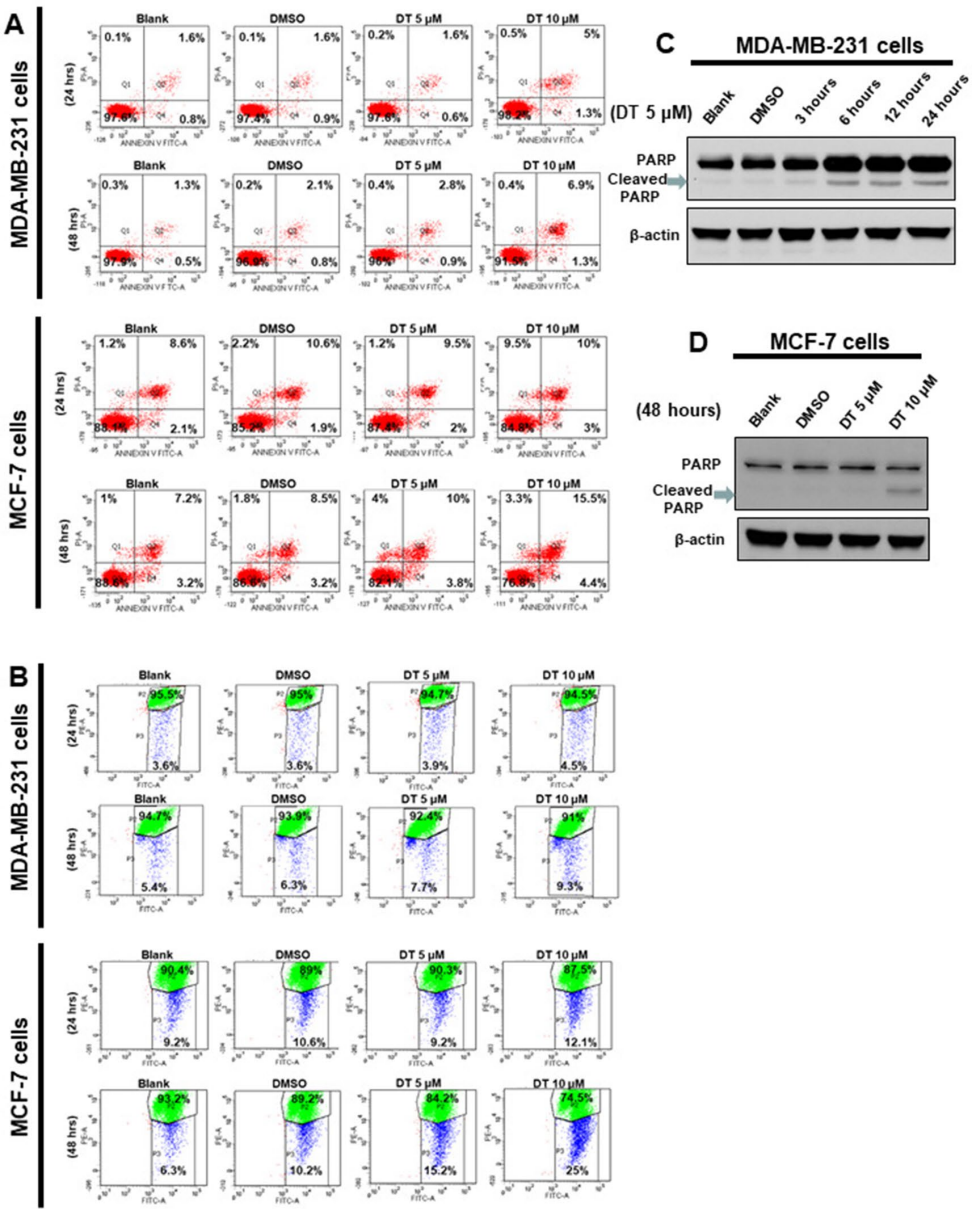
Dihydroisotanshinone I (DT) induces apoptosis in breast cancer cells. MCF-7 cells or MDA-MB-231 cells were treated without or with indicated compounds for 24–48h. Cell apoptosis was detected by flow cytometry with annexin-V-FITC/PI dual staining or mitoscreen JC-1 staining. **(A)** For annexin-V-FITC/PI dual staining, the representative histograms of flow cytometric analysis using double staining with annexin-V-FITC (FITC-A) and PI (PI-A). **(B)** For mitoscreen JC-1 staining, dot Plots revealing depolarization of mitochondria in treated indicated breast cancer cells. The percentage of events in the upper gate (P2) and lower gate (P3) represent population of treated indicated breast cancer cells having normal and depolarized mitochondria respectively. **(C**, **D)** Total cell extracts of MDA-MB-231 cells **(C)** or MCF-7 cells **(D)** were harvested from cells treated with DMSO or indicated concentrations of DT for indicated hours. The protein was immunoblotted with polyclonal antibodies specific for PARP. β-actin was used as an internal loading control.

In previous studies, we found that DT inhibited the proliferation of cancer cells through apoptosis (Lin et al., 2017; Wu et al., 2017b). We hope to investigate the role of apoptosis in DT treatment for breast cancer cells. For this purpose, MDA-MB-231 cells and MCF-7 cells were treated with the indicated compounds for 24 to 48 h and were subsequently analyzed for apoptosis by using flow cytometry with Annexin V/PI dual staining. The results demonstrated that 5–10 μM DT induced apoptosis in MDA-MB-231 cells in a time- and dose-dependent manner after 24 and 48 h. However, DT only induced significant apoptosis in MCF-7 cells after 48 h and not 24 h (**Figure 4A**). Moreover, 5–10 μM DT induced the mitochondrial depolarization of MDA-MB-231 cells in a time- and dose-dependent manner after 24 and 48 h, as shown by using the JC-1 staining assay. However, DT only induced significant mitochondrial depolarization of MCF-7 cells after 48 h but not 24 h (**Figure 4B**). As DT induced the most pronounced apoptotic effect on MDA-MB-231 cells, even at the lowest concentration (5 μM), we also discovered that 5 μM DT could upregulate the expression of a critical apoptotic protein, cleaved PARP, in MDA-MB-231 cells in a time-dependent manner (between 3 and 24 h) (**Figure 4C**). In addition, we observed that 10 μM DT upregulated the expression of cleaved PARP in MCF-7 cells after 48 h (**Figure 4D**). These results suggested that apoptosis was one of the modes of cell death induced by DT in breast cancer cells.

### DT Induces the Ferroptosis of Breast Cancer Cells Through Downregulating GPX4 Protein Expression

**Figure 5:**
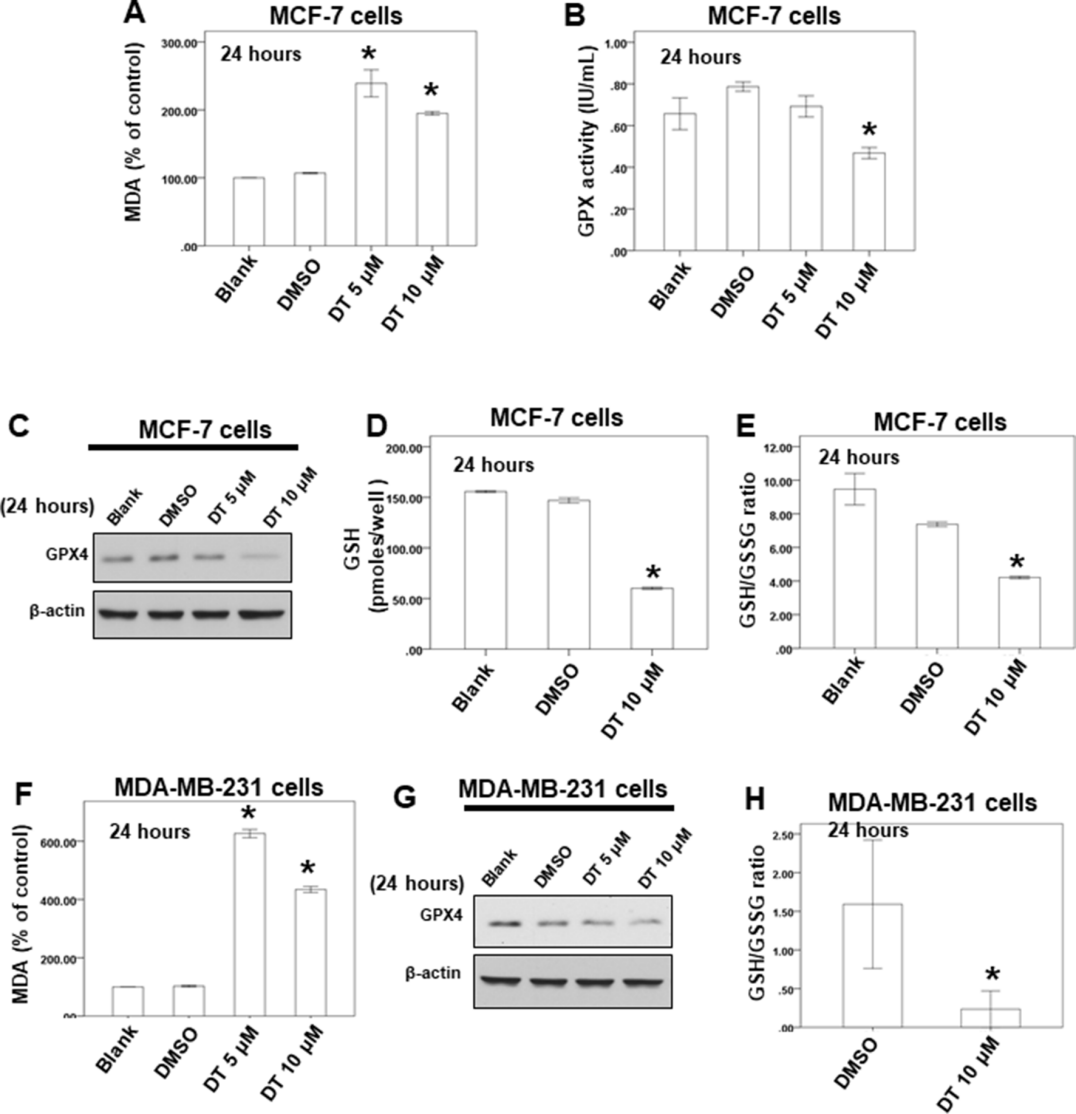
DT induced ferroptosis in breast cancer cells in vitro. **(A**, **F)** For MDA assay, MCF-7 cells **(A)** or MDA-MB-231 cells **(F)** were treated with DMSO or indicated drugs for 24 hours. Total cell extracts of indicate breast cancer cells was collected and analyzed by MDA assay kit. **(B)** For GPX activity, MCF-7 cells were treated with DMSO or indicated drugs for 24 hours. Total cell extract was collected and analyzed by GPX activity assay kit. **(C**, **G)** Total cell extracts of MCF-7 cells **(C)** or MDA-MB-231 cells **(G)** were harvested from cells treated with DMSO or indicated concentrations of DT for 24 hours. The protein was immunoblotted with polyclonal antibodies specific for GPX4. *β*-actin was used as an internal loading control. **(D**, **E**, **H)** For GSH and GSSG level, indicated breast cancer cells were treated with DMSO or indicated drugs for 24 hours. Total cell extract was collected and analyzed by GSH and GSSG assay kit. (Error bars=mean±S.E.M. Asterisks (*) mark samples significantly different from DMSO group with p < 0.05).

Our data showed that 10 μM DT treatment inhibited the growth of MCF-7 and MDA-MB-231 breast cancer cells, with inhibition rates of approximately 47.142% and 62.55%, respectively, after 24 h. However, DT treatment did not induce significant apoptosis in MCF-7 cells after 24 h (**Figure 4A**). Moreover, 10 μM DT treatment induced apoptosis in only approximately 6.3% of the MDA-MB-231 cells after 24 h. These results suggested that another type of cell death may be induced by 10 µM DT within 24. Of the various forms of nonapoptotic cell death, ferroptosis plays a major role in numerous diseases, including cancer (Linkermann et al., 2014; Galluzzi et al., 2015; Yang and Stockwell, 2016). GPX4 is an important regulator of ferroptosis (Yang et al., 2014; Seibt et al., 2019). To study whether the inhibition of cell proliferation induced by DT was associated with ferroptosis, we examined the mechanism of the effect of DT on breast cancer cells. Lipid peroxidation is a crucial process during ferroptosis (Yang and Stockwell, 2016). MDA, a natural biproduct of lipid peroxidation, is often used as a marker for lipid peroxidation. After treatment with the indicated compounds for 24 h, our results revealed that 5–10 μM DT significantly increased the MDA level in both MCF-7 and MDA-MB-231 cells (**Figures 5A, F**).

GSH and GSSG constitute a critical cellular antioxidant system and provide a reducing environment to reduce oxidative species. GPX4 is an essential regulator of ferroptosis. The loss of GPX4 can cause a drastic increase in GSSG, leading to a decrease in the GSH/GSSG ratio. Subsequently, ferroptosis is driven by the loss of activity of GPX4 (Yang et al., 2014; Canli et al., 2016; Yang and Stockwell, 2016; Seibt et al., 2019). First, we discovered that 10 μM DT could reduce the GPX activity of MCF-7 cells after 24 h (**Figure 5B**). Second, our results revealed that 10 μM DT significantly inhibited the protein expression of GPX4 in MCF-7 cells after 24 h (**Figure 5C**). Notably, 10 μM DT caused a drastic increase in GSSG and a decrease in the GSH/GSSG ratio in MCF-7 cells after 24 h (**Figures 5D, E**). Moreover, DT had the same inhibitory effect on the protein expression of GPX4 and decreased the GSH/GSSG ratio in MDA-MB-231 cells, even at 10 μM (**Figures 5G, H**). These results suggested that ferroptosis was one of the modes of cell death induced by 10 μM DT in breast cancer cells.

### *In Vivo* Effect of DT on Xenograft Nude Mouse Model

**Figure 6:**
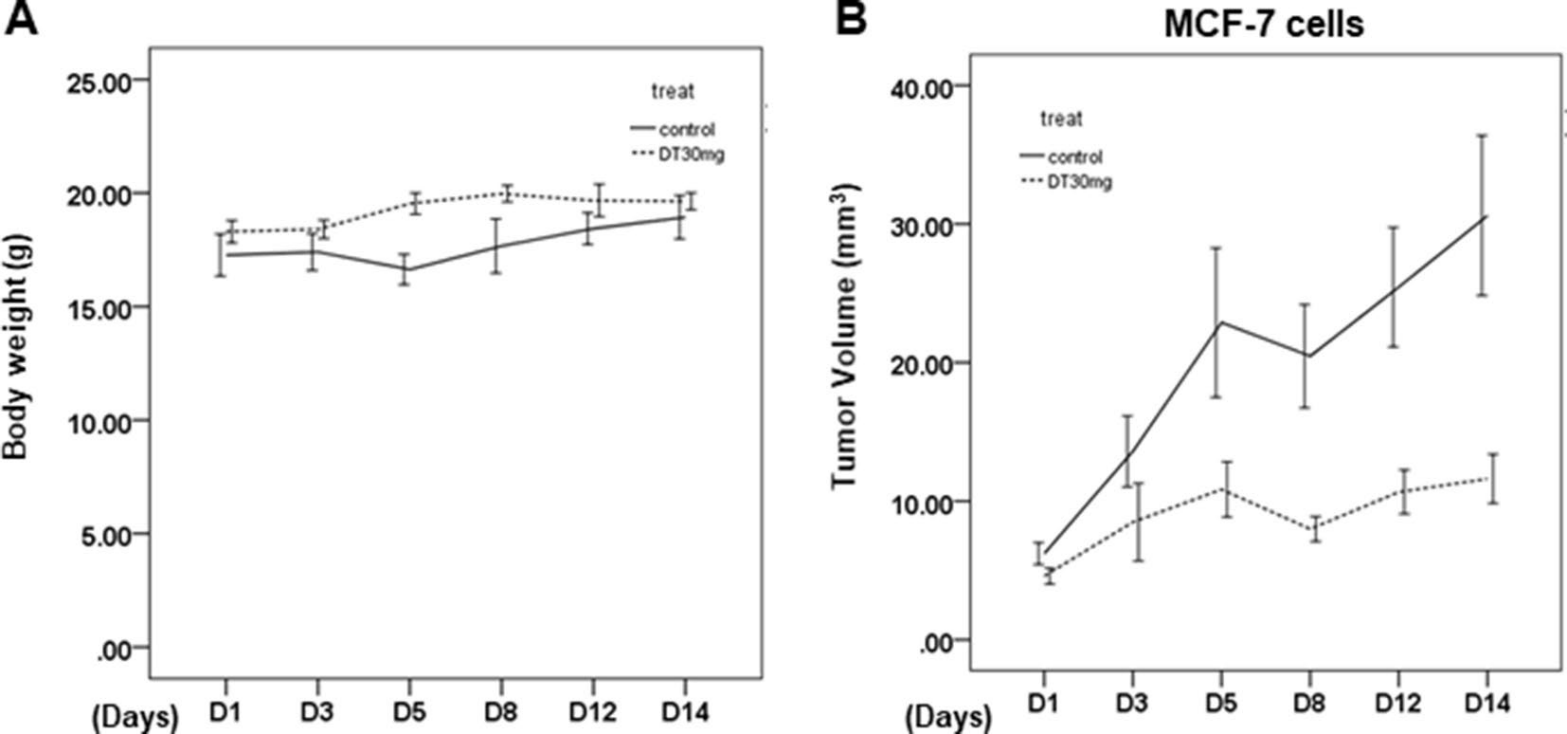
The *in vivo* effect of dihydroisotanshinone I (DT) on xenografted animal model. **(A)** Average mice weights with every 2-day injection of vehicle/DT over a time course of 2 weeks. **(B)** Average tumor volume of mice injected with either vehicle (DMSO) or DT (30 mg/kg, n = 5 per group). (Error bars = mean ± S.E.M.).

To investigate the effects of DT *in vivo*, we used female mice xenografted with a tumor as a model of cancer. A previous study showed that tanshinone IIA (50 mg/kg, IP), with a similar structure to DT, inhibited the tumor growth in the MDA-MB-231 xenograft mouse model (Li et al., 2015). In our previous study, DT treatment (30 mg/kg, IP) also significantly inhibited the final tumor volume in mice xenografted with HCT-116 cells (colon cancer) (Lin et al., 2017). From the XTT, we found that DT had a stronger inhibitory effect on the growth of MCF-7 cells compared with MDA-MB-231 cells (**Figure 3**). For these reasons, we treated mice xenografted with MCF-7 cells with 30 mg/kg DT. After 2 weeks of DT treatment (30 mg/kg, IP), there was no significant alteration in either the activity or the body weight of the mice, and no mice died (**Figure 6A**). Moreover, we discovered that DT treatment (30 mg/kg, IP) significantly limited the final tumor volume, by approximately 70%, after 2 weeks (**Figure 6B**). These results suggested that DT treatment induced only limited adverse events in mice, validating our data from the cell lines.

## Discussion

In a previous study, Lee et al. found that the use of traditional Chinese medicine was associated with a lower risk of mortality in patients with breast cancer, and some herbs, including Bai Hua She She Cao, Ban Zhi Lian, and Huang Qi, had a stronger effect on the reduction of mortality (Lee et al., 2014). However, no other research or evidence has explained or supported the finding. In this study, we used a real-world database to investigate the clinical effect of danshen on the survival rate of patients with breast cancer in a nationwide cohort study in Taiwan. Next, we used cellular and animal experiments to discover the molecular mechanism of DT, a compound purified from danshen, in breast cancer. However, some limitations of this cohort study should be noted. For the mode of danshen prescription, we discovered that TID (three times a day) was most commonly used frequency of prescription for danshen (**Table S1**). For the distribution of total danshen dosage (**Table S2**), we found the numbers of theses group (total dosage> = 336g, 168g< = total dosage <336g, 84g< = Total dosage < 168g, and Total dosage <84g) were 251, 266, 349, and 20,472 respectively. However, because the NHI program provided reimbursements for both western’s medicine and Chinese medicine, these patients usually used both western’s medicine and Chinese medicine for the treatment of cancer in Taiwan. Some reports showed that danshen combination with western medicine can affect the circulation (Xu et al., 2018; Ren et al., 2019). Some physicians may hold the prescription of danshen during operation, chemotherapy, or other anticancer treatments. For this reason, it is difficult to discover the consistent mode of danshen treatment from NHIRD. To overcome the problem, we investigated the total expose dose of danshen to discover the effect of danshen on breast cancer patients depending on the previous literature (Yang et al., 2015). Next, although we used the treatment of taxane to distinguish the population of patients with breast cancer, there was no clear information on the stages of breast cancer from the NHIRD. In contrast, because the number of patients in the groups treated for more than 28 days or treated with more than 84 g was too low to be divided into different groups (e.g., higher dose or longer period of treatment) in our analysis (during 2000 to 2010), we could not determine the protective effect of higher doses or a longer period of treatment of danshen at different stages of breast cancer.

Next, the combined use of Chinese medicine therapy with western medicine is prevalent in Taiwan. The main purpose of these combining Chinese medicine with systemic cancer therapy is to diminish the systemic cancer treatment-related adverse effects (Wang et al., 2014). In our study, the clinical outcome of danshen combined with different anti-breast cancer medications remains unclear. Moreover, the study cohort included patients based on the International Classification of Diseases, Ninth Revision, Clinical Modification codes (code: 174, malignant neoplasm of breast) from the NHIRD; these patients comprised all receptor statuses of breast cancers, including ER-positive and triple-negative cancers. We also could not determine the protective effort of danshen on patients with different receptor status in this study. Through this cohort study, we determined that the use of danshen could prolong the survival rate of patients with breast cancer in Taiwan. However, more rigorous, randomized, double-blind, and placebo-controlled trials are necessary to confirm this finding.

Several studies have demonstrated that tanshinone IIA has an anticancer effect on both ER-positive and ER-negative breast cancers (Wang et al., 2005; Su and Lin, 2008; Su et al., 2012; Yan et al., 2012; Lin et al., 2013; Fu et al., 2014; Li et al., 2015; Li and Lai, 2017; Lin et al., 2018; Lv et al., 2018; Wu et al., 2018). Several studies have revealed that tanshinone I also has an anti-breast cancer effect (Nizamutdinova et al., 2008a; Nizamutdinova et al., 2008b; Gong et al., 2012; Wang et al., 2015). From our data, we also observed that DT has a more marked inhibitory effect than SA at 5–10 μM on the proliferation of both ER-positive and ER-negative breast cancers cells (**Figures 3B, C**). These data suggested that danshen may have a clinical protective effect for patients with both ER-positive and ER-negative breast cancer owing to the constituent abietane diterpenes. Moreover, some studies have demonstrated that, in combination with tanshinone IIA, danshen could enhance the chemosensitivity of breast cancer cells to chemotherapy and even overcome drug-resistant cancer (Li and Lai, 2017; Lin et al., 2018; Lv et al., 2018; Wu et al., 2018). Further research to investigate the clinical protective effect of danshen combined with other breast cancer medication is necessary.

As shown by our results, 5–10 μM DT significantly increased the level of MDA, a marker of lipid peroxidation, in breast cancer cells after 24 h (**Figures 5A, F**). However, only 10 μM DT could reduce the level of GPX4 after 24 h (**Figures 5C, G**). In a recent report, Gao et al. demonstrated that mitochondria have a major role in cysteine deprivation-induced ferroptosis but not in GPX4 inhibition-induced ferroptosis (Gao et al., 2019). Their results suggested that cysteine deprivation leads to hyperpolarization of the mitochondrial membrane and lipid peroxide accumulation. We discovered that 10 μM DT could induce mitochondrial depolarization after 24 to 48 h, but that 5 μM DT did not induce mitochondrial membrane depolarization after 24 h in both the breast cancer cell lines tested. These results suggested that the changes in mitochondrial membrane potential or cysteine deprivation may play a role in lipid peroxide accumulation observed after 5 μM DT treatment for 24 h.

Collectively, the information from the real-world NHIRD represents a novel model to demonstrate that danshen exerted protective efforts in patients with breast cancer in Taiwan. As shown in *in vitro* and *in vivo* studies, DT inhibits the proliferation of breast cancer cells through apoptosis and ferroptosis. DT may be a novel anti-breast cancer agent; therefore, further prospective randomized studies are warranted to validate this finding.

## Data Availability Statement

The datasets generated for this study are available on request to the corresponding author.

## Ethics Statement

This study adhered to strict confidentiality guidelines according to regulations for personal electronic data protection and was approved by the Ethics Review Board of Chang Gung Memorial Hospital, Chia-Yi Branch, Taiwan (201601433B1). All procedures involving mouse xenograft model were approved by Animal Care and Use Committee (Approval number 2015060201) of Chang Gung Memory Hospital.

## Author Contributions

C-YW conceived the idea and designed experiments and wrote manuscript. Y-SL, Y-CS, Y-YT, Y-HY, Y-YL, L-HS, H-TL, Y-CC, Y-HuW and Y-HeW performed the experiments; C-NL, G-HC, M-ST, I-YL, Y-HuW and F-CK analyzed the data; D-CC revised the manuscript. All authors reviewed and approved the final version.

## Funding

This work was supported by grants CMRPG6F0662 and CMRPG6H0161 from the Chang Gung Memorial Hospital, and MOST105-2320-B-182-006-MY3 from the Ministry of Science and Technology to C-YW.

## Conflict of Interest

The authors declare that the research was conducted in the absence of any commercial or financial relationships that could be construed as a potential conflict of interest.

The reviewer, YH, declared a shared affiliation, with no collaboration, with one of the authors, C-NL, to the handling editor at the time of review.

## Abbreviations

DT, dihydroisotanshinone I; GPX4, Glutathione peroxidase 4; GSH, reduced glutathione; GSSG, oxidized glutathione; MDA, Malondialdehyde; NHI, National Health Insurance; NHIRD, National Health Insurance Research Database; SA, Salvianolic acid B.

**Chemical compounds studied in this article:** Dihydroisotanshinone I (PubChem CID:89406); Salvianolic acid B (PubChem CID: 6451084); Oxaliplatin (PubChem CID: 5310940); Gemcitabine hydrochloride (PubChem CID: 60749); 5-Fluorouracil (PubChem CID:3385).

## Supplementary Material

Table S1 The frequency of danshen prescription.

Table S2 The distribution of total danshen dosage.
