# Supplementary figures and images for: Danshen Improves Survival of Patients With Breast Cancer and Dihydroisotanshinone I Induces Ferroptosis and Apoptosis of Breast Cancer Cells
Source: Front Pharmacol. 2019 Oct 31;10:1226. doi: 10.3389/fphar.2019.01226 (PMC6836808; doi:10.3389/fphar.2019.01226)

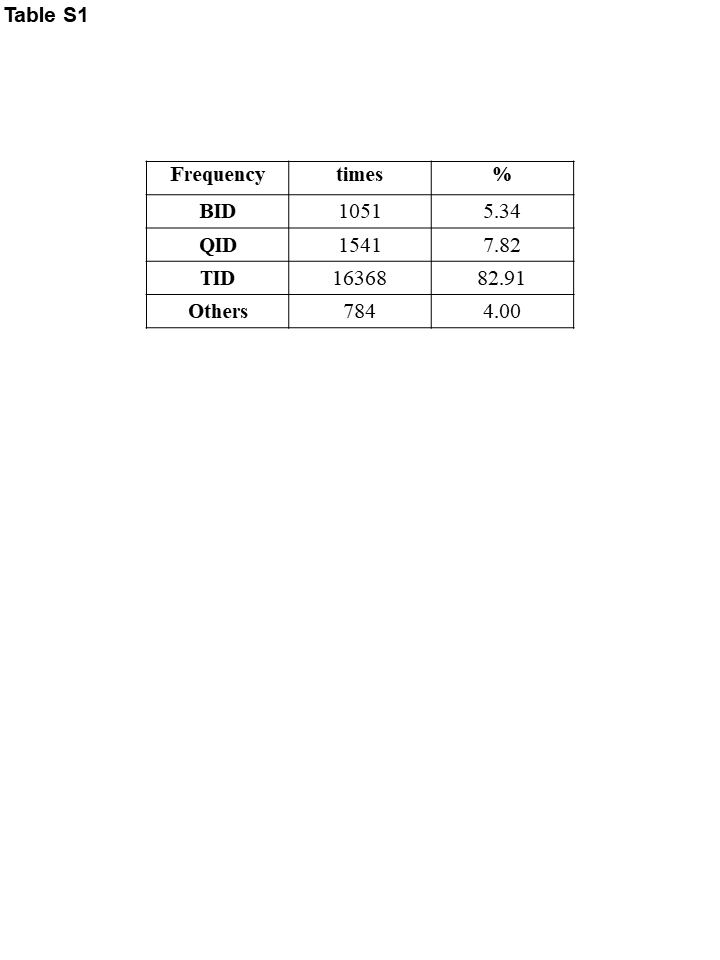

Supplement: Table S1 — The frequency of danshen prescription. [file Image_1.tif]

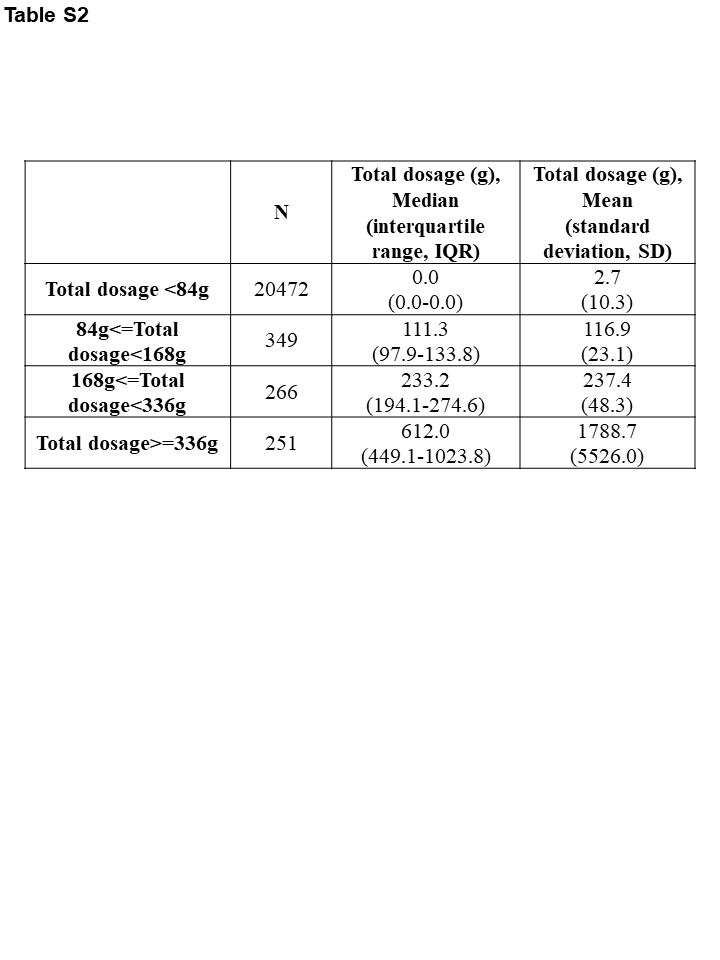

Supplement: Table S2 — The distribution of total danshen dosage. [file Image_2.tif]
